# Costal osteomyelitis due to *Bartonella* *henselae* in a 10-year-old girl

**DOI:** 10.5194/jbji-6-171-2021

**Published:** 2021-05-20

**Authors:** Arnaud Salmon-Rousseau, Christelle Auvray, Quentin Besset, Claire Briandet, Claire Desplantes, Pascal Chavanet

**Affiliations:** 1 Infectious Diseases Department, Dijon University Hospital, Dijon, France; 2 Laboratory of Virology, François Mitterrand University Hospital, Dijon, France; 3 Department of Pediatric Onco-Hematology, Dijon University Hospital, Dijon, France

## Abstract

*Bartonella henselae* is the bacterial agent responsible for cat scratch disease. This infection is frequently the cause of localized lymphadenitis in children. It is also sometimes responsible for endocarditis, encephalitis, hepatic peliosis and in rare cases osteomyelitis. We describe the second known case of unifocal thoracic osteomyelitis in a 10-year-old child.

## 1 Introduction

Cat scratch disease is the most common zoonotic disease, affecting children and young adults in 80 % of cases (Mirouse et al., 2015). The first French cases were reported in 1950 by Robert Debré, who described the presence of spontaneously resolving adenopathies in the drainage area following cat scratches (Debre et al., 1950).

The so-called classic clinical form manifests itself as a single or single-site, unilateral, inflammatory, and sensitive lymphadenopathy. Atypical forms have been described with systemic expressions depending on the immune status of the host: examples include Parinaud's oculo-glandular syndrome, hepato-splenic abscess, endocarditis and encephalitis etc. (Leclainche and Bourrillon, 1996; Carithers, 1985). Bartonella osteoarticular infections are rare, and in fact the prevalence of these bone manifestations lies between 0.2 % and 0.3 % (Hajjaji et al., 2007; Maman et al., 2007) and affects mostly children; 75 % of cases are unifocal (Zellali et al., 2019), and the infection is usually localized in the spine (Zellali et al., 2019). The rib cage is rarely the site of such infections. There are a total of six cases of multifocal osteomyelitis with thoracic involvement and only one case of unifocal thoracic osteomyelitis in the literature.

Here, we report the second case of a child hospitalized for a suspected thoracic tumor that was finally found to be cat scratch disease, and we provide a review of the literature on osteoarticular *B. henselae* infections in the pediatric population.

## 2 Method

We consulted the PubMed database to perform the present literature review.

We included systematic reviews, journals and case reports published in English since the first case was found in 1952.

We retained only cases reported for children, so all patients older than 18 years were excluded from the study.

The terms used in the search database were as follows: cat scratch disease, bone, bone infection, bone joint infection, bartonella, bartonellosis.

## 3 Case study

A 10-year-old girl presented with fever, diarrhea and diffuse muscle pain. Treatment with non-steroidal anti-inflammatory drugs and paracetamol was initiated by the treating physician, but the symptoms persisted and the patient developed bone pain in the sacroiliac, left thigh and left costal areas. She was hospitalized on the 10th day of clinical evolution, in a context of altered general condition and a weight loss equivalent to - 3.5 % of total body weight. The clinical examination was unremarkable and did not reveal lymphadenopathy or hepato-splenomegaly. The first blood test showed white blood cells at 15.57 G/L, CRP of 150 mg/L and sterile blood cultures.

**Figure 1:**
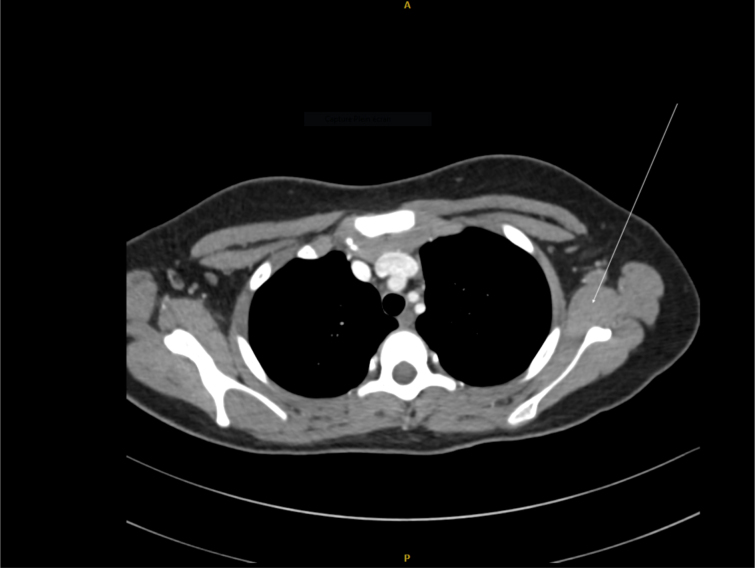
Thoracic CT scan. The arrow shows the left axillary ganglion. Origin and source of radiology image: Department of Radiology CHU Dijon.

The thoracic–abdominal–pelvic CT scan showed moderate hepato-splenomegaly, a 20×23 mm left axillary ganglion (Fig. 1) and retro-pectoral lymph nodes larger than 1 cm. There were no abnormalities in the bone window.

A technetium-99m bone scan revealed increased uptake in the left midrib.

Treatment with paracetamol and naproxen 10 mg/kg/d reduced the fever and allowed the patient to return home, and the diagnostic retained was then chronic aseptic osteomyelitis.

**Figure 2:**
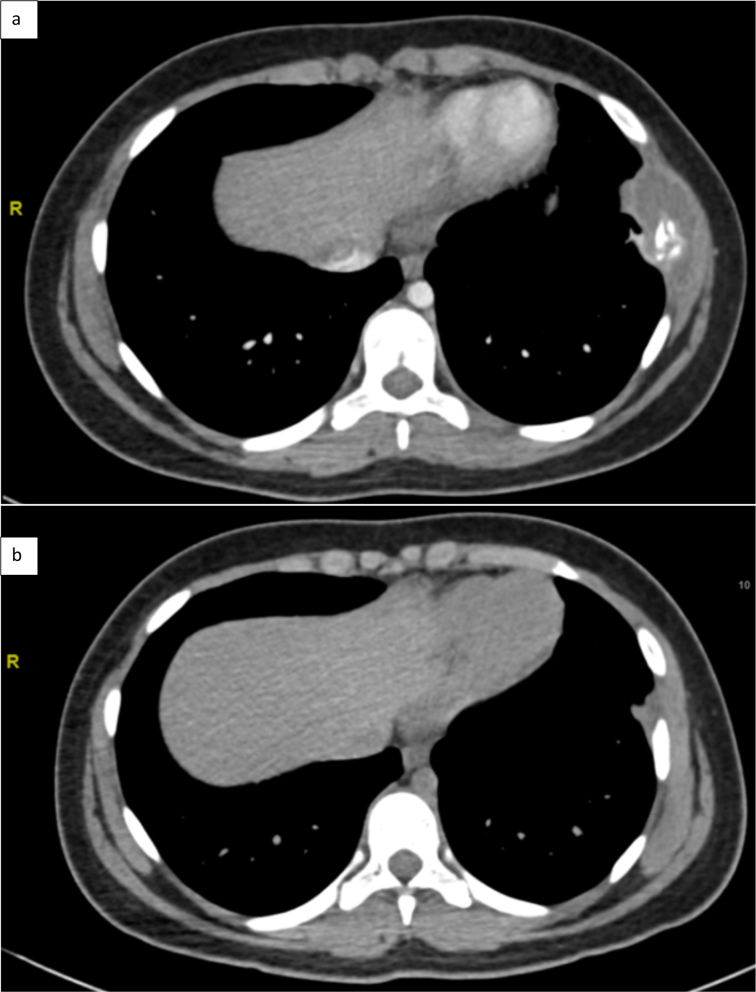
Thoracic CT scan **(a)** at diagnosis **(b)** end of treatment. Origin and source of radiology images: Department of Radiology CHU Dijon.

One month later, the child was again experiencing pain in the left costal area. Bioassay results showed hyperleukocytosis (10.7 G/L) and an elevated CRP level (37 mg/L). A new CT scan showed a single bone lesion on the anterior arch of the seventh left rib with a blown aspect and cortical lysis surrounded by a tissue sleeve. The lesion was 46 mm high, 64 mm deep and 34 mm wide, with a necrotic-looking tissue component (Fig. 2a).

The child was referred to the university hospital for a suspected chest tumor.

A surgical biopsy of the middle arch of the left seventh rib was performed, and the intraoperative findings revealed a purulent fluid with false membranes, revealing a lytic lesion of the rib.

Direct examination of pus after Gram staining found no bacteria. A few colonies of *Staphylococcus lugdunensis* were found after e days of culture. This result was considered as contamination of the sample taken by the surgeon.

Anatomo-pathological examination revealed granulomatous tissue, punctuated by small foci of necrosis surrounded by polymorphic inflammatory elements, rich in histiocytes and CD68 + macrophages around the foci. The periodic-acid–Schiff, Gram and Ziehl–Neelson stains were negative.

An interview with the girl's parents revealed the presence of kittens in the home. Further tests including *Bartonella henselae* serology returned with an IgG titre of 1/1280 (IFI technique), for a positivity threshold of 1/320. *Bartonella henselae* polymerase chain reaction (PCR) on whole blood was negative and 16S PCR on biopsy tissue was positive for *Bartonella sp.*

Treatment with a combination of azithromycin and rifampicin for 6 weeks completely improved the symptoms: lasting apyrexia, disappearance of pain in a few days and a weight gain of 3 kg at mid-treatment.

The anti-*Bartonella* IgG titre at the end of treatment was 1/640, and the chest CT scan revealed that the peri-costal collection had resolved and there was a favorable evolution of the bone lesion (Fig. 2b).

Follow-up at 6 months from the end of treatment found the girl in good general condition with no recurrence of fever or pain.

**Table 1:** Osteomyelitis associated with cat scratch disease, cases reported. Sex: M = male; F = female. Age: y = year. Trunk: C = cervical; T = thoracic; L = lumbar; S = sacrum. Blank field: no data; fever in degrees Celsius.

Year	Authors	Sex/age	The bone	Portions	Lymphadenopathy	Fever	Serology	Antibiotics	Duration of therapy(day)
			Column	Limbs					
1954	Adams and Hindman (1954)	M/5y	pelvic bone		cervical/inguinal	38.1			
1959	Collipp and Koch (1959)	M/4y	pelvic bone	hip	cervical		+	Penicillin/doxycycline	9/15
1969	Carithers et al. (1969)	F/6y		Ve metatarsus	axilla	38.1		Erythromycin/chloramphenicol	15/10
1983	Carithers (1983)	M/2y	sternum			38.4		No treatment	
1985	Johnson et al. (1985)	M/18y	rachis		cervical				
1987	Muszynski et al. (1987)	M/2y	frontal bone		inguinal	38		Surgical treatment then cloxacillin	14
1987	Walterspiel and Nimityongskul (1987)	F/7y		humerus				dicloxacillin	?
1989	Shanon et al. (1989)	M/11y	L4		axilla	38.2		Penicillin-M	?
1990	Karpathios et al. (1990)	F/8y	T5					No treatment	
1992	Cohen-Abbo et al. (1992)	M/9y	fronto-parietal bone /T12-L1		cervical	40		Cefalexin/erythromycin/gentamicin	?
1992	Larsen and Patrick (1992)	F/10y	skull/L4L5		mandibular/cervical/inguinal			Ceftriaxone then oxacillin	?
1993	Fretzayas et al. (1993)	M/12y	rachis					Penicillin A	?
1994	Bernini et al. (1994)	F/5y	T9			38.3			
1994	Koranyi (1994)	M/4y	rachis		axillar				
1994	Waldvogel et al. (1994)	M/9y	parietal bone		cervical	40	IgG 1/1024	Penicillin-M/fosfomycin	?/10
1996	Gallemore and Worley (1996)	M/6y	rib/rachis	femur	retro-pharyngeal		+		
1996	Hopkins et al. (1996)	M/6y	L2		retro-pharyngeal	yes	+		
1998	Berg et al. (1998)	F/1y	skull		no		IgG 1/2048		
1998	Keret et al. (1998)	M/9y		metacarpo-phalangeal				Clindamycin then Cotrimoxazole/rifampicin	21 14
1998	LaRow et al. (1998)	M/10y	pubic/iliac crest		cervical	40	IgG 1/1014	Cefazolin	14
1998	Ratner et al. (1998)	M/10y	iliac wing/ischium			yes	IgG 1/1024		
1999	Hulzebos et al. (1999)	F/10y	L2		cervical	40	IgG 1/600	Ciprofloxacin/fifampicin	42
1999	Maggiore et al. (1999)	F/7y		humerus	inguinal		IgG 1/256	Erythromycin	10
1999	Robson et al. (1999)	F/9y	T9		inguinal	40.8	IgG 1/2048	Cotrimoxazole/gentamicin	84/8
2000	Liapi-Adamidou et al. (2000)	F/7,5y	8th rib/L2/sacro-iliac /hip	knee	mesenteric	40	IgG 1/1024	cefotaxime	15
2000	Ruess et al. (2000)	F/12y	rachis		sub-mandibular /axillar	40	IgG 1/8000	erythromycin	21
2001	Fretzayas et al. (2001)	F/9y	rib rachis pelvis		axillar/epithroclear		+		
2001	Modi et al. (2001)	F/4y	parietal bone 11th 12th Rib, cup, iliac crest			40	IgG 1/1024	Surgical treatment Azithromycin/rifampicin	28
2002	Del Santo et al. (2002)	F/2y	L4-L5			40	+	Azithromycin	?
2002	Prybis et al. (2002)	M/6y		femur			+		
2003	Mirakhur et al. (2003)	F/3y	Orbital osteomelitis		retroperitoneal	40	IgG 1/64	Amoxicillin-clavulanic acid/rifampicin	35
2003	Rolain et al. (2003)	M/10y	rachis		axillar		+	Doxycyclin/ciprofloxacin/macrolide	?
2003	Sakellaris et al. (2003)	F/6y	10th rib		cervical	39.8	IgG 1/8192	Clarithromycin/gentamicin	12/8
2004	Ledina et al. (2004)	F/22 months		humerus	cervical	37.2	IgG 1/128	Azithromycin/cotrimoxazole	20
2005	Abdel-Haq et al. (2005)	M/5y	T4-T5-T7		axillar	yes		Surgical treatment then Cotrimoxazole/clarithromycin	5
2005	Hipp et al. (2005)	M/10y	sacrum ilium	femur		39	IgG 1/4096	Azithromycine	21
2005	Hipp et al. (2005)	F/3y		tibia	cervical	40	IgG 1/512	Azithromycin	25
2006	De Kort et al. (2006)	F/9y	sacrolumbar spine	elbow, collarbone, humerus				Cotrimoxazol/rifampicin	99
2006	Vermeulen et al. (2006)	F/9y	cervical rachis			39.6	+	Amoxicillin-clavulanic acid	21
2007	Hussain and Rathore (2007)	M/3y	T9		cervical, submandibular	40	+	Macrolide/rifampicin	42

**Table 1:** Continued.

Year	Authors	Sex/age	The bone	Portions	Lymphadenopathy	Fever	Serology	Antibiotics	Duration of therapy (day)
			Column	Limbs					
2007	Kodama et al. (2007)	F/11y	T3 L4L5	femur		39	IgG 1/1024	Azithromycin/doxycycline	28
2007	Rozmanic et al. (2007)	M/11y	8th rib, T8, iliac bone		inguinal	40	IgG 1/8192	Azithromycin/rifampicin	42
2008	Ridder-Schröter et al. (2008)	F/12y		humerus	axilla/ epithroclear	38.3	IgG 1/1024	Clarithomycin then clindamycin/rifampicin	10 11
2009	Tasher et al. (2009)	M/5y	C1-C4-C5		cervical, submandibular	38.5		Surgical treatment then Azithromycine/rifampicin	?
2010	Kossiva et al. (2010)	F/13y	hip, acetabulum		cervical		IgG 1/1024	Ceftriaxone	10
2011	Boggs and Fisher (2011)	M/11y		cubitus			+	Azitrhomycin	21
2012	Al-Rahawan et al. (2012)	M/7y	T6-T8				IgG 1/512	Azithromycin	14
2013	Dusser et al. (2013)	F/13y	hip, sacrum	humerus, femur tibia		40	IgG 1/512	Azithromycin	28
2015	Lafenetre et al. (2015)	F/13y	T2T3 L4L5	femur	submandibular		neg	Cotrimoxazole/rifampicin	21
2015	Knafl et al. (2015)	M/18y	T7		cervical	40	IgG 1/10 000	Azithromycin/rifampicin	21
2015	Mirouse et al. (2015)	M/14y	C1-C2		supracondylar		négative	Amoxicillin-clavulanic acid/ciprofloxacin	90
2016	Dornbos et al. (2016)	F/5y	T8-T11		inguinal	40	IgG 1/128	Azithromycin then Doxycycline/rifampicin	5 46/25
2017	Harry et al. (2018)	F/9y	sternum, rib, pelvis	hip		38.8	IgG 1/512	Azithromycine	28
2017	Harry et al. (2018)	F/3y	skull, eye socket, T4 T12		cervical	39.4	IgG 1/1024	Azithromycine	28
2017	Rafferty et al. (2017)	M/5y	C7T1			38.6	IgG 1/1024	Ciprofloxacin/rifampicin	42
2018	Akbari et al. (2018)	M/7y	C2-C4		cervical		IgG 1/1024	Azithromycin/rifampicin	42
2018	Aoki et al. (2018)	F/2y	pelvic bone	femur	cervical		IgG 1/1024		
2018	Donà et al. (2018)	F/12a	temporo-parietal bone		cervical, submandibular			Azithromycin then Cotrimoxaxol/rifampicin	15 84
2018	Karski et al. (2018)	F/1.5y		radius			IgG 1/320	Surgical treatment	
2018	Mathews et al. (2018)	F/12y		elbow, hip			IgG 1/1024	Azithromycin	42
2018	Rafee and English (2018)	F/3y	frontal bone		pre-auricular/inguinal			Azithromycin/rifampicin	42
2018	Zellali et al. (2019)	M/3y	pelvic bone, S4–S5		inguinal			Cefamandol/azithromycin then Amoxicillin-clavulanic acid/rifampicin	15 42

## 4 Discussion

The diagnosis of osteoarticular infections in children is difficult. The incidence of all these infections is low, estimated to be between 7.1 and 22 per 100 000 population (Mitha et al., 2015; Grammatico-Guillon et al., 2013), and they can affect all parts of the skeleton even if they are predominately found in the lower limbs: 75 %–80 % of cases (Vial and Chiavassa-Gandois, 2012). The main bacteria that cause these infections are group *B streptococcus* in children under 3 months of age, *Kingella kingae* between 6 months and 4 years of age, and *Staphylococcus aureus* at all ages (Ferroni et al., 2013).

The main differential diagnosis for rapidly progressing bone disease is a neoplastic process (Massei et al., 2000).

Cat scratch disease affects an estimated 40 000 people worldwide, with 80 % of cases occurring in people under 18 years of age (Mirouse et al., 2015). The prevalence of osteomyelitis in *Bartonella henselae* varies between 0.2 % and 0.3 % (Hajjaji et al., 2007; Maman et al., 2007). Spinal injury is the most common manifestation (42 % of cases) and multifocal injury is seen in 25 % of cases (Zellali et al., 2019).

Our review of the literature identified 62 cases of *B. henselae* osteomyelitis in children published since 1954; only 7 cases included costal involvement (Table 1). The typical clinical picture is a child under 10 years of age with fever, cervical polyadenopathy and an average weight loss of 4.5 kg (Table 1). The scratch of a cat was observed in 20 out of 62 cases without necessarily being in the territory of the adenopathy (Table 1). Rare musculoskeletal manifestations (Maman et al., 2007) were reported for 30 out of 62 children, of which 20 % were arthralgia.

Biological examinations did not provide enough data to suggest a particular diagnosis: leukocytes were higher than 10 G/L for 21 out of 62 children. Non-discriminating inflammatory syndrome was generally found, with average CRP median of 20 mg/L ( < 5–111 mg/mL).

Standard radiology was performed for 17 children, focusing on the painful segment. Osteolysis was sometimes found and in some cases associated with sclerosis or even a periosteal reaction within an infiltration of the surrounding soft tissues (Carithers, 1983; Johnson et al., 1985; Mazur-Melewska et al., 2015; Rohr et al., 2012). CT scans (performed on 25 children) confirmed bone destruction. MRI (31 children) was mostly used to evaluate the extent of lesions and whether they involved the bone marrow, adjacent tissues and the nervous system. Bone scintigraphy (23 children) offered the advantage of mapping the body, which revealed foci at a distance from the osteoarticular apparatus or detected abscesses on the liver and/or spleen in 15 out of 23 and 11 out of 23 children respectively. The potential of the PET scan has not yet been evaluated in this context.

Serology and molecular biology (polymerase chain reaction) techniques were used on tissue samples for microbiological diagnosis (Dusser et al., 2013; Hansmann et al., 2005). A total of 46 children were seropositive, and the anti-*Bartonella* IgG titre was greater than 1/512 for 26 of them. Only two children tested negative. *Bartonella* PCR was performed on 17 tissue samples and was positive on all samples; there were no false negatives in this series. The sensitivity of PCR analyses is estimated at 60 %–75 %, with high specificity allowing species diagnosis between *Bartonella* (Hansmann et al., 2005; Ratner et al., 1998; Eglantin et al., 2008).

Due to the rarity of osteoarticular forms of cat scratch disease, there are no defined antibiotic protocols. Macrolides were used for 52.0 % of children, 22 of whom received azithromycin. Beta-lactam antibiotics were also used in 34.8 % of children, fluoroquinolones in 7.6 % and doxycycline in 6 %.

When dual therapy was initiated (42.6 % of children), rifampicin was associated in 29.0 % and aminoglycosides in 13.6 %. Other combinations were either with Fosfomycin, chloramphenicol or cotrimoxazole.

In our case, the choice of antibiotic therapy (azithromycin and rifampicin) was motivated by their low minimal inhibitory concentrations reported in the literature (azithromycin 0.006–0.015 µ g/mL, rifampicin 0.03–0.06 µ g/mL), but also by their intracellular activity (Rolain et al., 2004; Bass et al., 1998).

The median duration of antibiotic therapy is 22 d (5–99 d).

Only 2 children (3.2 %) did not receive antibiotics, and 4 (6.4 %) laminectomy surgeries were performed.

## 5 Conclusions

*Bartonella* osteoarticular infections are rare in children, but should nonetheless be considered when a quickly progressing bone lesion is observed, a fortiori if there are signs of infection and there has been contact with animals, especially cats. *Bartonella henselae* serology should be carried out systematically in these cases, and close collaboration with the bacteriology laboratory should make it feasible to obtain a prompt diagnosis.

## Data Availability

No data sets were used in this article.
